# Astrocytes deficient in circadian clock gene *Bmal1* show enhanced activation responses to amyloid-beta pathology without changing plaque burden

**DOI:** 10.1038/s41598-022-05862-z

**Published:** 2022-02-02

**Authors:** Celia A. McKee, Jiyeon Lee, Yuqi Cai, Takashi Saito, Takaomi Saido, Erik S. Musiek

**Affiliations:** 1 Department of Neurology and Center On Biological Rhythms And Sleep, Washington University in St. Louis School of Medicine, St. Louis, MO USA; 2 Department of Neurocognitive Science, Institute of Brain Science, Nagoya City University Graduate School of Medical Sciences, Nagoya, Aichi Japan; 3 Laboratory for Proteolytic Neuroscience, RIKEN Center for Brain Science, Wako-shi, Saitama, Japan; 4 Present Address: Ann Romney Center for Neurologic Diseases, Department of Neurology, Brigham and Women’s Hospital, Harvard Medical School, Boston, MA USA

**Keywords:** Circadian mechanisms, Alzheimer's disease, Astrocyte

## Abstract

An emerging link between circadian clock function and neurodegeneration has indicated a critical role for the molecular clock in brain health. We previously reported that deletion of the core circadian clock gene *Bmal1* abrogates clock function and induces cell-autonomous astrocyte activation. Regulation of astrocyte activation has important implications for protein aggregation, inflammation, and neuronal survival in neurodegenerative conditions such as Alzheimer's disease (AD). Here, we investigated how astrocyte activation induced by *Bmal1* deletion regulates astrocyte gene expression, amyloid-beta (Aβ) plaque-associated activation, and plaque deposition. To address these questions, we crossed astrocyte-specific *Bmal1* knockout mice (Aldh1l1-Cre^ERT2^;Bmal1^fl/fl^, termed BMAL1 aKO), to the APP/PS1-21 and the APP^NL-G-F^ models of Aβ accumulation. Transcriptomic profiling showed that BMAL1 aKO induced a unique transcriptional profile affecting genes involved in both the generation and elimination of Aβ. BMAL1 aKO mice showed exacerbated astrocyte activation around Aβ plaques and altered gene expression. However, this astrogliosis did not affect plaque accumulation or neuronal dystrophy in either model. Our results demonstrate that the striking astrocyte activation induced by *Bmal1* knockout does not influence Aβ deposition, which indicates that the effect of astrocyte activation on plaque pathology in general is highly dependent on the molecular mechanism of activation.

## Introduction

The pathological hallmarks of Alzheimer’s Disease (AD)—the toxic accumulation of amyloid beta (Aβ) plaques and of hyperphosphorylated tau protein into neurofibrillary tangles—have been known to be accompanied by activated glia for over 100 years^1^. However, therapeutics aimed at targeting glial functions in AD have yet to gain traction in the clinical world, mostly because the roles of glia in Aβ or tau aggregation are still poorly understood. The amyloid cascade hypothesis of AD states that Aβ is likely the initiating factor in the neuroinflammatory cascade that leads to tau accumulation, neuronal loss, and eventually cognitive decline^2–5^. Despite setbacks in anti-Aβ clinical trials^6^, the amyloid cascade hypothesis is still widely employed in research and clinical settings^7,8^, and many treatment strategies are in development that target Aβ accumulation^9^. Thus, exploring roles for glia in preventing plaque formation and in clearing Aβ may provide a new avenue for therapeutic targets.

The influence of astrocyte activation in AD still remains to be elucidated: it is unclear whether astrocyte activation primarily exacerbates neuroinflammation and hastens pathogenesis, or whether it tips the balance towards beneficial functions in clearing Aβ and aiding neuronal survival^10^. These opposing possibilities are reflected in recent literature targeting astrocytes in AD. Several studies have associated astrocyte activation with increased plaque load^11–13^, while others have associated astrocyte activation with decreased plaque load^14,15^. These reports make it clear that the various contexts and transcriptional profiles of activated astrocytes participate in disease processes in very different ways. Clearly, there is still a need to define astrocyte genes that either reduce or accelerate Aβ pathology, and then to explore how those genes are expressed in different reactive states.

One set of genes that controls astrocyte functions is the molecular circadian clock. The circadian clock involves transcriptional-translational feedback loops that regulate rhythmic gene expression on a roughly 24-h basis. The positive arm of the core clock is made up of heterodimers composed of BMAL1 and either CLOCK or NPAS2, while the negative arm consists of PER, CRY, and REV-ERB proteins^16–18^. Together, these clock proteins regulate clock-dependent genes such that roughly 43% of protein-coding transcripts in mice exhibit daily rhythmicity^19^ and this number increases to more than 80% in primates^20^. Due to the wide range of genes controlled by the molecular clock, it is not surprising that the clock interacts with disease processes: AD symptoms include circadian disruption in both sleep–wake cycle and general activity^21–24^. Aβ and other disease pathologies also affect molecular clock rhythms through modification and degradation of clock genes and proteins^25,26^. Thus, unraveling the connection between clock-controlled gene expression and AD progression may reveal pathways amenable to targeting for AD treatment.

An important aspect of the circadian clock is that it is regulated in a tissue- and cell type-specific manner^19^. An emerging literature has shown that the circadian clock in astrocytes modulates gliotransmission and transmitter levels^27–31^, behavioral rhythms^27,29,30,32^, metabolism^33^, and neuroinflammation^34^. We have recently reported the robust cell-autonomous activation of astrocytes by deletion of the clock gene *Bmal1*, which induces several activation and inflammatory markers^34^. Moreover, we have also shown that deletion of *Bmal1* globally disrupts Aβ interstitial fluid rhythms and exacerbates Aβ plaque burden in the hippocampus^35^, though the role of astrocyte BMAL1 in this process is unclear. Taken together, these findings suggest that the astrocyte circadian clock could regulate activation and downstream genes that may influence Aβ generation, deposition, and clearance. In this study, we deleted *Bmal1* specifically in astrocytes and explored how Aβ pathology was altered in two AD mouse models. This work sheds light on the many astrocyte clock-controlled genes relevant to AD and their impact on Aβ accumulation.

## Materials and methods

### Mice

All mouse experiments were conducted in accordance with protocols approved by the Washington University Institutional Animal Care and Use Committee (IACUC), which is accredited by AAALAC. All experiments were conducted in accordance with all relevant guidelines and regulations and were performed in a manner consistent with ARRIVE guidelines (https://arriveguidelines.org). *Aldh1L1-Cre*^*ERT2*^, and *Bmal1*^*fl/fl*^ mice were obtained from The Jackson Laboratory (Bar Harbor, ME; stock #031008 and 007668, respectively) and were bred at Washington University. *APP/PS1-21* mice^36^ were a gift from Dr. Mathias Jucker (Tubingen, Germany). *APP*^*NL-G-F/NL-G-F*^ mice^37^ were crossed with *Bmal1*^*fl/fl*^; *Aldh1L1-Cre*^*ERT2*+^ to generate *APP*^*NL-G-F/wt*^ mice. All cohorts of mice were mixed sex and consisted of Cre+ and Cre- littermates from several breeding cages. All mice were maintained on a C57BL\6 background and housed under 12-h light/12-h dark conditions. All *Aldh1L1-Cre*^*ERT2*^ (Cre- and Cre+) were given tamoxifen (Sigma, dissolved in corn oil, 2 mg/mouse/day for 5 days) by oral gavage at 2 months of age to induce *Bmal1* astrocyte-specific deletion. All mice were group housed with food and water available ad libitum*.* BMAL1 aKO mice were harvested at 5–7 months of age (1 week, 4 weeks, and 12 weeks post-tamoxifen). *APP/PS1-21* mice were harvested at 4 months of age (2 months post-tamoxifen) and *APP*^*NL-G-F/wt*^ mice were harvested at 9.5 months of age (7.5 months post-tamoxifen).

### Immunohistochemistry

Mice were anesthetized with an i.p. injection of Fatal Plus pentobarbital and then perfused with ice cold PBS-heparin. Tissues were harvested and brains were post-fixed in 4% paraformaldehyde overnight at 4 °C. Brains were then washed with PBS before being cryoprotected in 30% sucrose in PBS at 4 °C. 50 µm sections were made on a Leica freezing microtome and stored in cryoprotectant solution (30% ethylene glycol, 15% sucrose, 15% phosphate buffer in ddH20) at − 20 °C. On the day of staining, sections were first briefly washed in PBS. For X34 plaque staining, sections were permeabilized in PBS containing 0.25% triton X-100 (PBSX) for 30 min at room temperature. X34 (stock solution 10 mM in DMSO) was diluted 1:1000 into staining buffer containing 60% PBS, 40% ethanol, and 0.02 N sodium hydroxide, and sections were incubated in X34 staining buffer for 20 min at room temperature. Sections were then washed in X34 wash buffer containing 60% PBS and 40% ethanol, and then washed in PBS. Following X34 plaque staining, sections were then blocked and permeabilized with 3% goat or donkey serum in PBSX and stained with the following antibodies: rabbit anti-GFAP (Dako Z0334, 1:5000), rat anti-LAMP2 (EMD Millipore MABC40, 1:1000), HJ3.4b anti-Aβ (lab of Dr. David Holtzman, 1:1000), goat anti-IBA1 (Abcam ab5076, 1:500), rabbit anti-Bmal1 (Novus Biologicals NB100-2288, 1:2000), Topro (Invitrogen T3605, 1:2000).

Sections were incubated in primary antibodies diluted in PBS containing 1% serum and 0.25% triton X-100 and stored on a shaker in the dark overnight at 4 °C. Sections were then washed several times in PBS and then incubated in secondary antibodies diluted 1:1000 in 0.25% triton X-100 for 1 h at room temperature. Sections were washed again in PBS. Sections were given final PBS washes and then mounted on slides with Prolong Gold.

For AT8 staining, frozen brain sections were washed three times with Tris-buffered saline (TBS) for 5 min followed by incubation in 0.3% hydrogen peroxide in TBS for 10 min at room temperature. Sections were washed three times with TBS for 5 min, blocked with 3% milk in 0.25% TBS-X (Triton X-100) for 30 min, and then incubated with biotinylated AT8 antibody (MN1020B; Thermo Fisher Scientific) in 1% milk-0.25% TBS-X at 4 °C overnight. After three washes with TBS for 5 min, the slices were incubated with ABC Elite (Vector PK-6100) in TBS for 1 h and were visualized using 3,30—diaminobenzidine tetrahydrochloride (DAB) (Chromogen).

### Imaging and analysis

For quantification of cortical and hippocampal pathology, slides were imaged on a Nikon Eclipse 80i epifluorescence microscope equipped with a Hamamatsu Orca-Flash 4.0 digital camera. Images were imported into Fiji (Fiji Is Just Image J) for percent area and BMAL1 expression analyses. For BMAL1 quantification within astrocytes, individual astrocyte nuclei were first identified within GFAP/Topro images. Astrocyte Topro nuclei were included if they were individually identifiable (not clustered) and were surrounded by GFAP staining. 15 nuclei per image were identified and then scored as either BMAL positive or negative by an observer blind to genotype. For AT8 tau quantification, stained sections were imaged using a NanoZoomer, and quantification was performed using Image J percent area and particle count functions. For 3D analysis in Imaris, z-stacks were taken on a Nikon A1R confocal at 20 × with a step size of 0.5um. Z-stacks were analyzed in Imaris: 3D surfaces were generated for Aβ plaques (X34, detail 0.2um, threshold 700, voxels above 2000um), astrocytes (GFAP, detail 0.2um, threshold 800, voxels above 50um), and dystrophic neurites (LAMP2, detail 0.3um, threshold 800, voxels above 2000um). Astrocyte-plaque contacts were quantified using a surface-surface colocalization function to generate a new surface from colocalizing voxels within the astrocyte and plaque surfaces, for which the volume was quantified as “astrocyte-plaque contact” and was normalized to the plaque volume per image. Plaque “shell” 3D surfaces were then created using an Imaris XTension function generated at the Washington University Center for Cellular Imaging. Plaque surfaces were dilated either 30 µm or 50 µm and new surfaces were generated as 30 µm shells and 50 µm shells. Astrocyte and dystrophic neurite volume within these shells was calculated using a surface-surface colocalization function that made a new surface from only colocalizing voxels between a plaque shell and surface of interest (astrocytes or dystrophic neurites).

### ELISA assays

Brain cortices were sequentially homogenized in PBS followed by 5 M guanidine. Sandwich ELISA was performed using antibodies provided by Dr. David Holtzman (Washington University in St. Louis School of Medicine). Half-well 96-well plates were coated with HJ7.4 (anti-Aβ37–42) at 4 °C overnight. Samples were diluted in sample buffer containing protease inhibitors and incubated on the ELISA plate overnight at 4 °C. Aβ42 was detected with a biotinylated HJ5.1 antibody (anti-Aβ13–28) followed by a streptavidin-poly-HRP-40 (Fitzgerald Industries). All ELISA assays were developed using Super Slow ELISA TMB (Sigma) and absorbance was read on a Bio-Tek Synergy 2 plate reader at 650 nm. Standard curves were prepared from synthetic human Aβ42 from rPeptide.

### Real-time quantitative PCR

For RNA extraction from tissue, cortices were placed in Trizol and homogenized using steel beads and a bullet blender (Next Advance). Chloroform was added, and after centrifugation aqueous phases were collected. RNA was extracted using a PureLink RNA Mini Kit (Life Technologies), and RNA concentrations were measured on a nanodrop spectrophotometer (Thermo Nanodrop 8000) before storing RNA at −80 °C. cDNA was prepared from RNA samples using a high-capacity reverse transcription kit (Applied Biosystems). Taqman probes (Applied Biosystems) were combined with cDNA samples and real-time quantitative PCR was run on either a StepOnePlus or QuantStudio 12 k Real-Time PCR thermocycler (Applied Biosystems). Data were analyzed using the delta-delta Ct method with *Actb* or *Gapdh* as housekeeping genes.

### Fluidigm micro-fluidic qPCR

For Fluidigm micro-fluidic qPCR analysis of gene expression, RNA and cDNA were prepared as above and sent with Taqman probes to the Washington University Genome Technology Access Center for high-throughput qPCR on a Fluidigm BioMark HD system. Data were analyzed as described above.

### RNA sequencing and analysis

RNA sequencing and analysis were performed by the Genome Technology Access Center at Washington University using their standard methods, which are summarized here. Sample RNA integrity was determined using a Tapestation and library preparation was performed with 10 ng of total RNA for samples with a Bioanalyzer RIN score greater than 8.0. ds-cDNA was prepared using the SMARTer Ultra Low RNA kit for Illumina Sequencing (Takara-Clontech) per manufacturer's protocol. cDNA was then fragmented using a Covaris E220 sonicator using peak incident power 18, duty factor 20%, cycles per burst 50 for 120 s. The cDNA was blunt ended, had an A base added to the 3' ends, and then had Illumina sequencing adapters ligated to the ends. Ligated fragments were then amplified for 12–15 cycles using primers incorporating unique dual index tags. The fragments for each sample were then pooled in an equimolar ratio and sequenced on an Illumina NovaSeq-6000 using 150 base pair paired end reads. Basecalls and demultiplexing was performed with Illumina’s RTA 1.9 software and the reads were aligned to the Mus musculus Ensembl release 76 GRCm38 primary assembly with STAR version 2.5.1a. Gene counts were quantitated with Subread:featureCount version 1.4.6-p5.

All gene counts were then imported into the R/Bioconductor package EdgeR and TMM normalization size factors were calculated to adjust the samples for differences in library size. Ribosomal genes were removed and only genes expressed greater than one count-per-million in at least four samples were kept for further analysis. The adjusted TMM size factors and the matrix of counts were then imported into the R/Bioconductor package Limma. Weighted likelihoods were then calculated for all samples and the count matrix was transformed to moderated log 2 counts-per-million with Limma’s voomWithQualityWeights. Differential expression analysis was then performed to analyze for differences between conditions and the results were filtered for only those genes with Benjamini–Hochberg false-discovery rate adjusted p-values less than or equal to 0.1.

### Software

**Figure 1:**
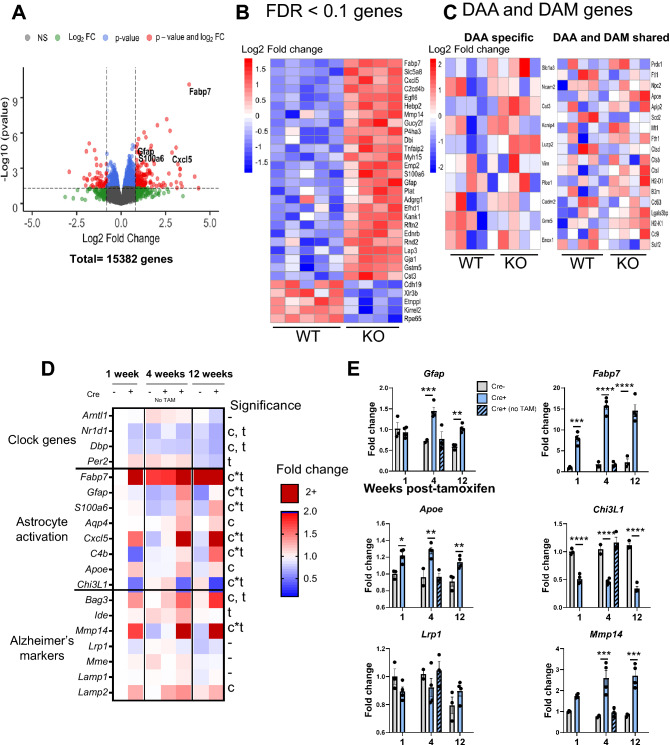
Brain transcriptomic analysis of astrocyte-specific *Bmal1* KO mice. (**A**–**C**) RNAseq analysis of frontal cortex from 5-month-old BMAL1 aKO mice and Cre- littermate controls (3 months post-tamoxifen) reveals differentially expressed genes defining clock-disrupted astrocytes (n = 5 WT, n = 4 BMAL1 aKO). (**A**)Volcano plot of differentially expressed genes in BMAL1 aKO (Log2 fold change cutoff = 1.2, -Log10pvalue cutoff = 0.05). (**B**) Heatmap of differentially expressed genes sorted by log2 fold change (FDR < 0.1). (**C**) DAA and DAM genes from Habib et al. 2020, only *Cst3* is significantly different (FDR < 0.1). (**D**,**E**) Fluidigm qPCR gene expression analysis of WT and BMAL1 aKO mice given tamoxifen for 5 days and harvested 1 week, 4 weeks, and 12 weeks later (n = 2–4 mice per group, mice 5.5–7 months-old). (**D**) Heatmap of genes involved in the circadian clock, astrocyte activation, and Alzheimer’s disease (AD). Two-way ANOVA analysis: c = significant main effect of Cre genotype, t = main effect of time post-tamoxifen, c*t = interaction effect of Cre and time, = no significance. (**E**) Individually plotted gene expression from D. * = p < 0.05, ** = p < 0.005, *** = p < 0.0005, **** = p < 0.0001 by two-way ANOVA with Sidak multiple comparisons test. Panels (**A**–**C**) was made using R Studio version 1.3.959 (https://www.rstudio.com), (**A**) using the Enhanced Volcano package, and (**B**,**C**) using Pretty Heatmaps package. (**D**) was generated using GraphPad Prism version 9.2 (https://www.graphpad.com).

Graphs and some heatmaps were generated using GraphPad Prism version 9.2 (https://www.graphpad.com). The volcano plot in Fig. 1 was generated using R Studio version 1.3.959, using the Enhanced Volcano package (https://www.rstudio.com), while heatmaps in Fig. 1B,C were generated using Pretty Heatmaps package. Image analysis was performed using Fiji version 2.1 (https://imagej.net/software/fiji) and Imaris 9 (https://imaris.oxinst.com).

## Results

In order to elaborate on the astrocyte activation state induced by *Bmal1* deletion that we have previously reported^34^ and to understand how disease-relevant genes are affected, we performed bulk RNA sequencing on cerebral cortex tissue from astrocyte-specific Bmal1 knockout mice (Aldh1l1-Cre^ERT2^;Bmal1^fl/fl^), termed BMAL1 aKO) and Cre-;Bmal1^fl/fl^ control littermates at 3 months post-tamoxifen treatment (5 months of age). Similar to our previous findings from global and brain-specific *Bmal1* KO mice, many genes associated with reactive astrocytosis were significantly upregulated, demonstrating that astrocyte BMAL1 regulates these genes in a cell-autonomous manner (Fig. 1B). Based on their reproducibility and fold change across experiments, we find that the most definitive markers of this activation state are *Fabp7, Gfap, Mmp14,* and *Cxcl5* (Fig. 1B,E).

We considered whether this activation state is transcriptionally similar to an Alzheimer’s disease-associated astrocyte profile as previously described by Habib et al.^38^, as this would provide clues to how clock-disrupted astrocytes may influence Alzheimer’s Disease. However, across genes shared by disease-associated astrocytes and microglia, we saw no significant differentially expressed genes in our RNA sequencing analysis other than *Cst3* (Fig. 1C), although by real-time qPCR we see a mild increase in *Apoe* expression in timecourse experiments (Fig. 1E). We also tested whether BMAL1 aKO fits an A1 or A2 reactive state but did not see any differentially expressed A1 or A2 genes, only the pan-reactive gene *Gfap* (data not shown), which matches our previous findings from brain-specific *Bmal1* KO mice^34^. Thus, our data suggests that the *Bmal1* astrocyte-specific knockout phenotype is a unique reactive state with an unknown contribution to brain health and disease.

To further validate these findings and explore the timecourse of the astrocyte activation phenotype, we treated BMAL1 aKO mice with tamoxifen and harvested mice at 1 week, 4 weeks, and 12 weeks post-tamoxifen. We then examined expression of genes involved in the circadian clock, the BMAL1 aKO astrocyte activation phenotype, and Alzheimer’s Disease. In this whole tissue analysis, the overall circadian clock expression was not remarkably different due to the fact that *Bmal1* was only deleted in astrocytes and much of its expression is in neurons, although *Nr1d1* and *Dbp* were significantly decreased in BMAL1 aKO Cre+ cortices at 1 week post-tamoxifen (Fig. 1D, for BMAL1 astrocyte-specific expression see Fig. 5A). For markers of the clock-disrupted astrocyte activation phenotype such as *Gfap*, *Fabp7*, *S100a6*, and *Cxcl5*, we found that the upregulation of these genes develops over time and is most striking at 3 months post-tamoxifen (Fig. 1D,E). Similarly, dysregulation of Alzheimer’s related genes such as *Apoe*, *Chi3l1*, and *Mmp14* also appeared most differentially expressed at 3 months (Fig. 1D,E). These data indicate that this activation state is stable, develops over time, and likely interacts with aging-associated processes.

Our time-course results also indicate that astrocyte BMAL1 regulates genes with conflicting reported effects on Aβ accumulation. For example, we have previously reported that knockout of the clock-controlled gene *Chi3l1* ameliorates Aβ pathology likely through facilitating Aβ phagocytosis by glia^39^. Here, we also found that BMAL1 aKO markedly reduces *Chi3l1* expression (Fig. 1E), indicating that Aβ pathology may be ameliorated by this astrocyte activation state. Similarly, *Mmp14*, an Aβ degrading enzyme^40^, was highly overexpressed in this model (Fig. 1E), suggesting increased capacity for Aβ degradation. However, we also found that *Apoe* expression is mildly increased (Fig. 1E). Increased *Apoe* expression in mouse AD models has been shown to promote amyloid plaque formation^41,42^, suggesting that BMAL1 aKO could lead to elevated plaque burden. In addition, some of the main genes involved in astrocyte Aβ uptake and degradation such as *Ide*^43–45^, *Mme*^43,46^, and *Lrp1*^47–49^ did not significantly change in BMAL1 aKO brain (Fig. 1D,E). As mentioned above, the BMAL1 aKO activation state also does not appear to overlap with the disease-associated astrocyte phenotype. Therefore, there may be conflicting effects on Aβ pathology induced by the clock-disrupted astrocyte.

**Figure 2:**
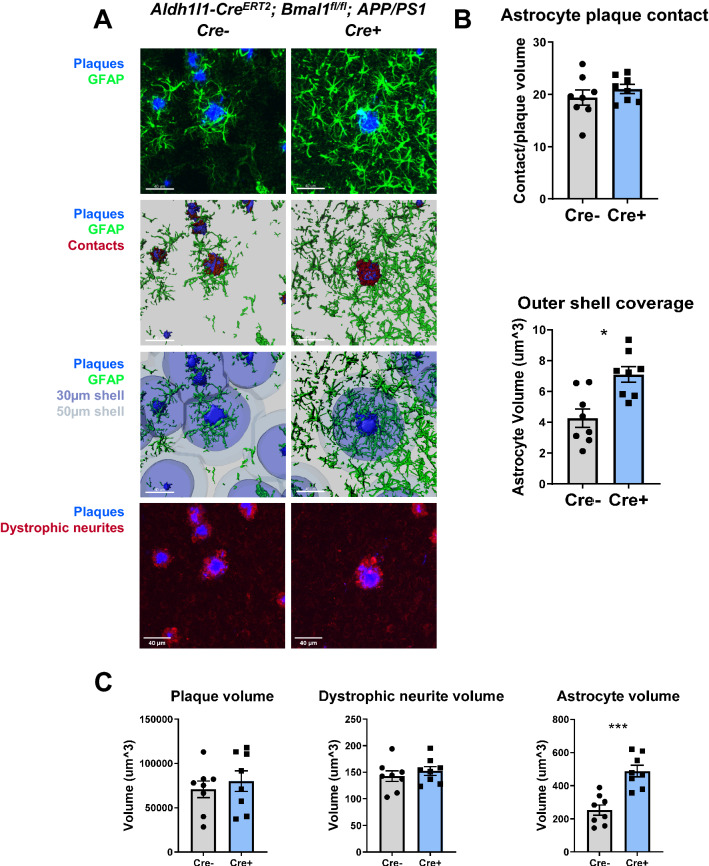
Astrocyte-specific *Bmal1* deletion increase peri-plaque astrocyte activation in APP/PS1 mice. (**A**) Confocal images and 3D renderings of cortical astrocytes and plaques from 4-month-old BMAL1 aKO; APP/PS1-21 mice and Cre- controls (scale bar = 40 µm). Plaques labeled in blue (X34) and astrocytes labeled in green (GFAP) with astrocyte-plaque contact represented by 3D overlap (red). Plaque shells rendered at 30 µm and 50 µm distance from plaque surface. (**B**) Quantification of astrocyte-plaque contact volume and astrocyte volume within the outer shell (50 µm from plaque surface). (**C**) Quantification of 3D plaque volume, dystrophic neurite volume (LAMP2) and astrocyte volume per 20X image. * = p < 0.05, *** = p < 0.0005 by T-test. Panel 1A was generated using Imaris version 9 (https://imaris.oxinst.com).

To understand how *Bmal1*-depleted astrocytes behave in the context of Aβ pathology, we examined astrocyte activation in the cortex of BMAL1 aKO mice crossed to the APP/PS1-21 model of Aβ-amyloidosis (Aldh1l1-Cre^ERT2^;Bmal1^fl/fl^;APP/PS1-21). APP/PS1-21 mice express human amyloid precursor protein and presenilin 1, both containing AD-associated mutations^36^. These mice develop fibrillar amyloid plaques beginning around 3 months of age. We treated mice with tamoxifen at 2 months of age to induce *Bmal1* deletion, then harvested mice at 4 months, when mild-moderate plaque burden is present. By using 3D reconstruction of X34-labelled fibrillar plaques and GFAP-expressing reactive astrocytes, we were able to observe and quantify astrocyte reactivity in relation to the plaque environment (Fig. 2A,B). We also quantified astrocyte contacts with plaques by measuring 3D overlap of GFAP and X34, as well as the volume of GFAP+ astrocyte staining within 30 and 50 µm “shells” generated around plaques (Fig. 2A,B). Our imaging analysis indicated that the overall GFAP+ astrocyte volume per image (Fig. 2C), as well as the GFAP+ astrocyte volume within the outer 50 µm shell (Fig. 2B) were increased in BMAL1 aKO;APP/PS1 mice, as compared to Cre-;APP/PS1 littermate controls. However, the 3D contact between the plaque surface and astrocytes was not significantly different (Fig. 2B). Notably, images were selected in this analysis such that the volume of Aβ plaques and peri-plaque dystrophic neurites per image were the same across *Bmal1* genotypes (Fig. 2C). These data indicate that plaque-associated astrocyte activation covers a wider area of tissue in the BMAL1 aKO brain, which may represent an interaction between BMAL1 aKO and Aβ in inducing astrocyte activation. However, BMAL1 aKO does not influence the physical interaction of astrocytes with plaques.

**Figure 3:**
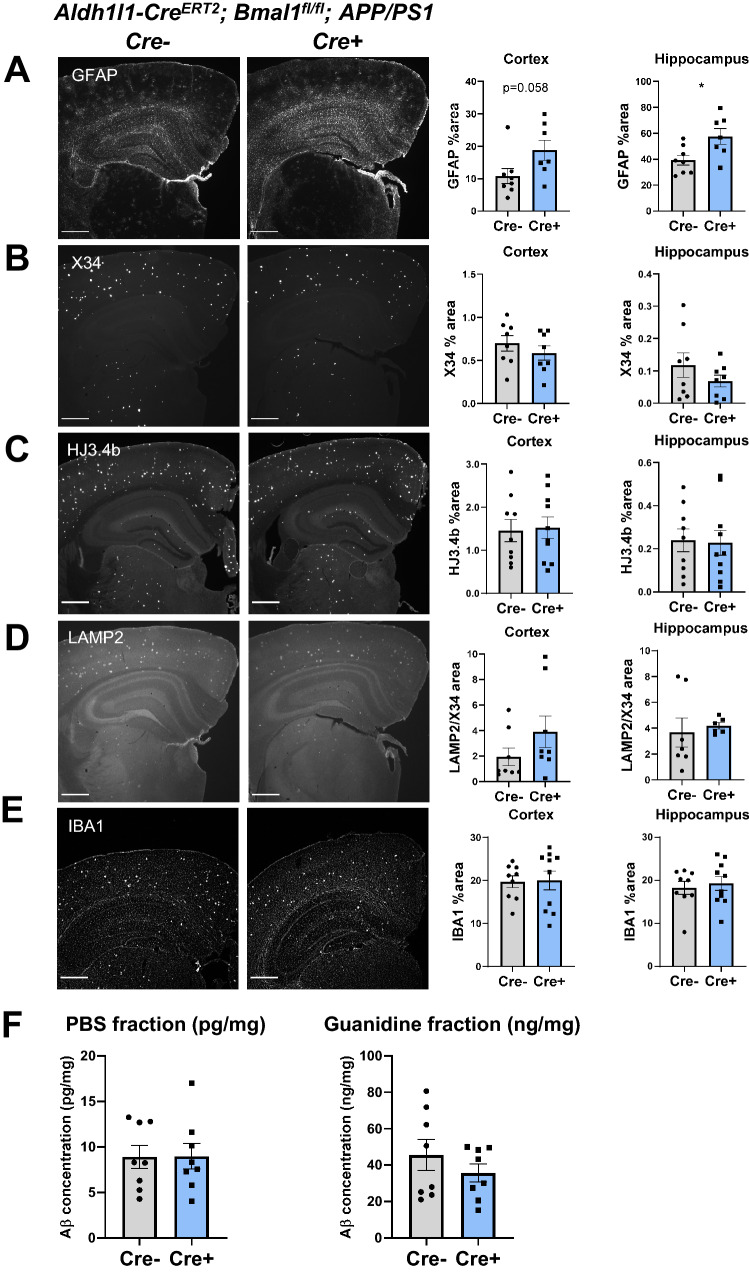
*Bmal1* deletion in astrocytes does not affect Aβ deposition in APP/PS1 mice. Images of brain sections (left) and percent area quantification (right) from 4-month-old old BMAL1 aKO; APP/PS1-21 mice and Cre- controls stained for GFAP (**A**), fibrillar Aβ (X34, **B**), total Aβ (HJ3.4b, **C**), dystrophic neurites (LAMP2, normalized to X34 area, **D**), and microglia (Iba1, **E**) (n = 6–10 mice per group, scale bars = 500 µm). (**F**) ELISA measurements of Aβ 1–42 in PBS- and Guanidine-soluble fractions from cortex (n = 8 mice per group). * = p < 0.05 by T-test.

To quantify the effect of BMAL1 aKO on Aβ deposition, we first performed imaging analysis of plaque burden in our BMAL1 aKO; APP/PS1 mice using the X34 stain for fibrillar plaques and an antibody to total Aβ (HJ3.4b). Surprisingly, while GFAP staining was increased in both the cortex and hippocampus for BMAL1 aKO mice (Fig. 3A), we found no significant difference in fibrillar plaque burden (X34+) or total Aβ plaques (HJ3.4b+) between Cre-;APP/PS1 and BMAL1 aKO APP/PS1 mice in either region (Fig. 3B,C). Dystrophic neurites (LAMP2) and microglia activation (IBA1) were also unchanged and mirrored plaque burden (Fig. 3D,E). Phosphorylated tau exhibited some mild signal in these mice, but this was far lower than a conventional P301S tau mouse and also did not show an effect of Cre genotype (Supp Fig. 1). In addition, we prepared PBS- and Guanidine-soluble fractions of brain lysates to quantify Aβ levels by sandwich ELISAs. Using an antibody for Aβ 37–42 (HJ7.4), we also found no difference in either PBS- or Guanidine-soluble fractions between Cre- and BMAL1 aKO APP/PS1 mice (Fig. 3F). These data indicate that despite a striking dysregulation of Alzheimer’s- and activation-associated genes and increased peri-plaque astrocyte activation, BMAL1 aKO does not influence Aβ plaque burden, dystrophic neurites, or microglial activation in the APP/PS1 model.

**Figure 4:**
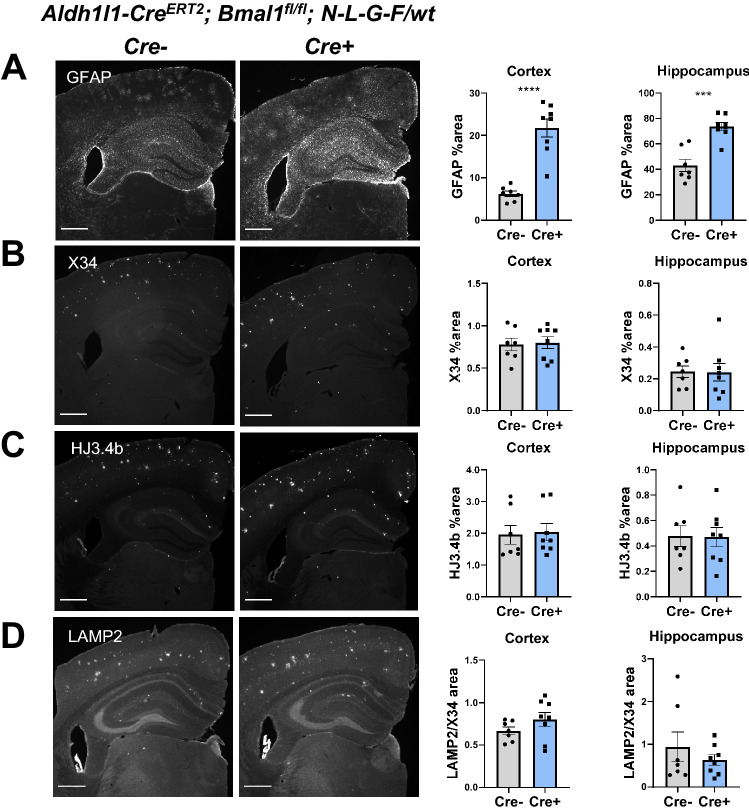
*Bmal1* deletion in astrocytes does not affect Aβ deposition in APP^NL-G-F/wt^. (**A**–**D**) Images of brain sections from 9.5-month-old BMAL1 aKO; APP^NL-G-F/WT^ knock-in mice and Cre- controls stained for GFAP(**A**), fibrillar Aβ (X34, **B**), total Aβ (HJ3.4b, **C**), and dystrophic neurites (LAMP2, **D**) (scale bars = 500 µm). Quantifications are percent area per cortex or hippocampus. n = 7–8 mice per group. *** = p < 0.0005, **** = p < 0.0001 by T-test.

We considered the possibility that 2 months of *Bmal1* deletion was an inadequate timeframe in which to observe an effect in the aggressive APP/PS1-21 model, as clock-disrupted astrocyte activation develops over time (Fig. 1). Thus, we sought to examine the effect of astrocyte *Bmal1* deletion in a slower model of amyloid deposition. The APP^N-L-G-F^ model is an APP knock-in mouse which has been shown to also produce Aβ plaques at a much slower rate when expressed in a hemizygous fashion^37^. As opposed to the 4-month time point for APP/PS1-21 mice, hemizygous APP^NL-G-F/wt^ mice take closer to 9 months to accumulate a significant number of Aβ plaques^37^. We hypothesized that the ability of activated astrocytes to influence Aβ deposition may depend on the rate of protein accumulation, as seen for astrocyte TFEB activation influencing a slower but not a more aggressive tau pathology model^50^. Thus, we generated BMAL1 aKO mice which were hemizygous for APP^N-L-G-F^ knock-in (Aldh1l1-Cre^ERT2^;Bmal1^fl/fl^;APP^NL-G-F/wt^) in order to investigate the role of clock-induced astrocyte activation in more slowly progressing Aβ pathology. After aging mice to 9.5 months, we harvested them for histopathology and gene expression analysis. Similar to the APP/PS1 model, BMAL1 aKO;APP^NL-G-F/wt^ mice had elevated GFAP expression in the hippocampus and cortex which extended beyond the immediate vicinity of the plaque (Fig. 4A). However, the BMAL1 aKO;APP^NL-G-F/wt^ mice also did not show a difference in X34-stained insoluble Aβ plaque burden, HJ3.4b-stained total Aβ plaque, or LAMP2 dystrophic neurite staining around plaques, as compared to Cre- littermates (Fig. 4B–D). Our results from these two Aβ pathology models indicate that *Bmal1* knockout in astrocytes increases peri-plaque astrocyte activation but does not influence Aβ plaque deposition or associated neuronal dystrophy.

**Figure 5:**
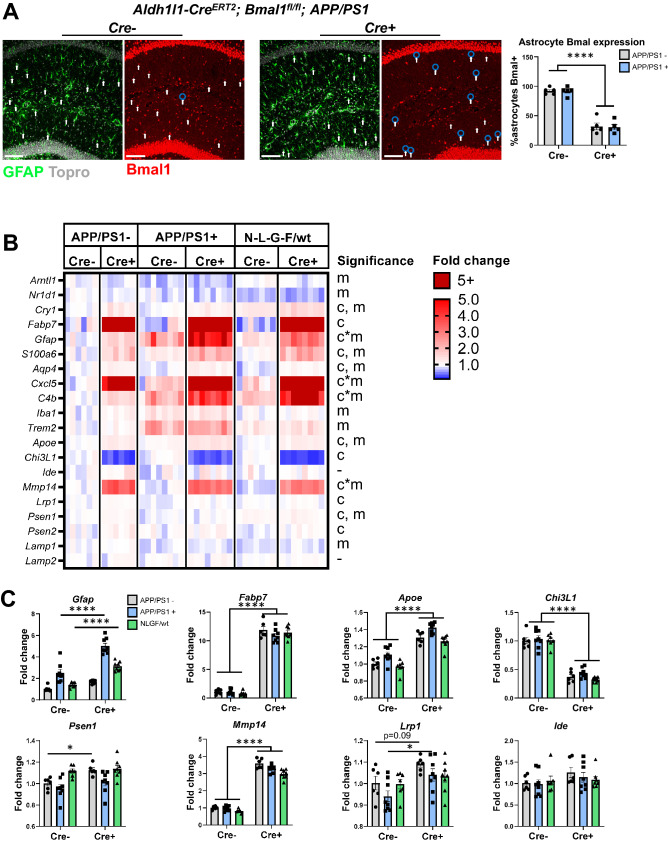
Astrocyte *Bmal1* regulates genes with conflicting effects on Aβ deposition. (**A**) Topro, GFAP, and BMAL1 staining in CA1 hippocampus of 4-month-old BMAL1 aKO; APP/PS1-21 mice and Cre- controls (scale bar = 100 µm). Arrows indicate astrocyte nuclei quantified as indicated by Topro nuclei surrounded by GFAP positivity. Blue circles indicate nuclei quantified as BMAL1 negative. Quantification of astrocytes counted as BMAL1- or BMAL1+ is shown on the right. n = 5 mice per group, **** = p < 0.0001 by two-way ANOVA with Sidak multiple comparisons test. (**B**) Heatmap of Fluidigm qPCR analysis of 20 genes involved in the circadian clock, glial activation, and Alzheimer’s Disease in cortex from Aldh1l1-Cre^ERT2^; Bmal1^fl/fl^ mice and Cre- controls with or without APP/PS1-21 or APP^NL-G-F/wt^ (n = 6–8 mice per group). Two-way ANOVA analysis: c = significant main effect of Cre genotype, m = main effect of Aβ model, c*m = interaction effect of cre and Aβ model, - = no significance (all p < 0.05). (**C**) Individually plotted genes from A. * = p < 0.05, ** = p < 0.005, *** = p < 0.0005 by two-way ANOVA with Sidak multiple comparisons test. Panel B was made using GraphPad Prism version 9.2 (https://www.graphpad.com).

We next decided to confirm that the BMAL1 aKO astrocyte activation state is retained long-term in these Aβ pathology models. It is possible that while *Bmal1* deletion initially activates astrocytes, the Aβ plaque environment is a stronger stimulus that shifts astrocytes to a DAA-like or other phenotype and overwhelms any effect of clock disruption on Aβ deposition. We first checked whether BMAL1 expression in our astrocyte knockout mice is still reduced in the plaque environment. We performed immunohistochemistry for BMAL1 since neurons and other cells express high levels of BMAL1 and make astrocyte-specific *Bmal1* knockout difficult to detect by whole tissue methods. By staining for BMAL1 in Cre- and Cre+ APP/PS1 mice, we observed that this knockout reduces BMAL1 within astrocytes by ~ 70% in both the wildtype and plaque-ladened brain (Fig. 5A). We examined gene expression using Fluidigm micro-fluidic qPCR on cortex tissue from Cre- and Cre+ mice with and without APP/PS1-21, as well as Cre- and Cre+ APP^NL-G-F/wt^ mice (Fig. 5B,C). Strikingly, the BMAL1 aKO astrocyte activation was retained in our Aβ plaque models, and for some genes, expression changes were indeed enhanced by the plaque environment. For example, *Fabp7* expression was consistently elevated in all BMAL1 aKO mice independent of Aβ status (Fig. 5B,C). *Chi3l1* expression levels also showed a main effect of BMAL1 aKO alone (Fig. 5B,C). On the other hand, *Gfap*, *C4b*, and *Cxcl5* showed an interaction effect for which the Aβ models further enhanced their upregulation in BMAL1 aKO mice (Fig. 5B,C). Alzheimer’s-associated gene expression again showed conflicting results: the Aβ-generating gamma-secretases *Psen1* and *Psen2* were weakly upregulated by BMAL1 aKO, mainly in the APP/PS1- littermate controls. *Lrp1* was also upregulated by BMAL1 aKO, but this was only significant in the APP/PS1+ mice. Regarding Aβ degrading enzymes, *Mmp14* increased in BMAL1 aKO but was not impacted by Aβ, while *Ide* showed no changes. *Apoe* also showed upregulation in response to BMAL1 aKO, and this was increased in the APP/PS1-21 mouse. Importantly, microglial genes such as *Iba1*, Cd68, *Trem2, Tlr2, Tlr4,* and *Tyrobp* only changed with Aβ model, mainly APP/PS1, and were not affected by *Bmal1* status (Fig. 5B,C, Supp Fig. 2). These observations demonstrate that astrocyte *Bmal1* deletion alters the astrocyte response to Aβ pathology, leading to synergistic dysregulation of certain BMAL1-regulated astrocyte genes. The involvement of BMAL1 in both Aβ-generating and Aβ-reducing processes also illustrates how astrocytes that exhibit an activated profile can still contribute a net-negative effect to Aβ deposition.

## Discussion

In its position as an essential core clock gene that regulates a wide variety of gene expression, *Bmal1* has the potential to determine cell-type specific responses to shifts in brain health. Here, we have demonstrated that *Bmal1* deletion dysregulates a range of astrocyte transcripts and induces activation, which is enhanced in Aβ plaque mouse models. However, BMAL1 aKO neither prevents nor exacerbates Aβ deposition. This curious observation indicates that it is critical to understand how specific astrocyte genes and functions are regulated in various activation states in order to interpret their effect on Aβ generation and degradation.

Previous literature has provided some insights into how astrocytes behave in the plaque-ladened brain and how they may influence Aβ accumulation. Most recently, it has been reported that disease-associated astrocytes (DAAs) in 5xFAD mice have a transcriptional profile that diverges from GFAP-expressing wildtype astrocytes^38^. DAAs express markers involved in endocytosis, the complement cascade, and aging, including a number of genes linked to Aβ accumulation. In our study, it is likely that some of these processes are altered in BMAL1 aKO, but the gene sets we have identified do not align with those in DAAs. However, it is also worth noting that some of the “wildtype” GFAP-high defining genes reported by Habib et al., such as *Gfap*, *Aqp4*, *Apoe*, and *Fabp7* are significantly elevated in BMAL1 aKO. Thus, *Bmal1* deletion induces an astrocyte activation state that may represent a ”wild-type” activated astrocyte, rather than the plaque-induced DAA profile.

Studies of astrocyte activation states in AD models have not yet definitively determined how activation impacts Aβ accumulation. Whether activated astrocytes ameliorate or exacerbate disease may depend on the context and transcriptional profile of their activation. For example, two studies have explored knockout of the critical astrocyte activation genes *Gfap* and/or *Vim* in APP/PS1 Δe9 mice^14,51^. While one study found that this method of decreasing astrocyte activation accelerated Aβ pathogenesis and neuritic dystrophy^14^, the other found no difference in Aβ plaque load, even though there were several transcriptional changes^51^. Another study involved deletion of the astrocyte water channel gene *Aqp4* and found increased plaque burden and synaptic protein damage associated with reduced astrocyte activation, though AQP4 presumably regulates glymphatic function as opposed to classical astrocyte activation^15^. These studies contrast with those that have found that activated astrocytes exacerbate disease: *Stat3* knockout or inhibition prevents astrocyte reactivity and decreases plaque load^11,12^, as does Clusterin overexpression^13^.

It is also worth noting that several astrocyte-expressed genes have been shown to specifically regulate Aβ accumulation, though these genes are not necessarily tied to typical astrocyte activation. For example, *Lrp1* expression in astrocytes regulates Aβ uptake and limits plaque formation^49^. It has been suggested that LRP1 shows strong expression in reactive astrocytes around plaques^52^, but expression of LRP1 in AD patient brains has yielded conflicting reports, some of which suggest that the overall expression of LRP1 is reduced in AD^53^. *Lrp1* is also not among the DAA genes^38^. Conversely, *Apoe* is primarily expressed in astrocytes and is among the genes induced in DAAs. APOE secreted from astrocytes may inhibit *Lrp1*-mediated Aβ uptake^54^, and generally promotes plaque accumulation^41^. In addition, considering that human APOE isoforms drastically differ in their risk for AD^55^, the overall levels of APOE potentially generated by reactive astrocytes may have various effects. Expressing the risk-associated Apoe4 isoform in mice increases Aβ accumulation and reactive astrocytes^56^, but *Apoe* isoforms do not appear to change expression as a result of astrocyte activation^57^. We also observed reduced expression of the AD-associated gene *Chi3l1*, which we expected to increase microglial activation and reduce plaque burden due to our previous report^39^ However, as these effects were not seen in this study, we suspect that greater suppression of *Chi3l1* over a longer time frame as seen for *Chi3l1* knockout mice is required to produce this reduction in plaque load. Thus, the relationship between astrocyte activation itself and the expression and function of genes which influence Aβ deposition is not straightforward.

So how might the circadian clock participate in AD? We have mostly focused on changes to Aβ-related genes and their effect on Aβ accumulation and neuronal dystrophy in astrocyte-specific *Bmal1* knockout, but there are several other clock functions that may alter the course of AD pathogenesis. We have previously reported that global *Bmal1* knockout elevates Aβ plaque burden in the hippocampus^35^. This was associated with altered soluble Aβ rhythms in the interstitial fluid, which may be most directly regulated by Aβ release from neurons rather than other brain cell types. Our results herein suggest that loss of astrocyte *Bmal1* does not explain the increase in plaques observed in global *Bmal1* KO mice. Thus, further investigating the contribution of the neuron-specific clock to Aβ accumulation may uncover one link between clock disruption and AD progression.

In addition, the astrocyte clock may be involved in other aspects of AD pathogenesis not investigated in this study. Rhythms in locomotor activity are at least partially controlled by the astrocyte clock^27,30,32^, and it is likely that activity rhythms affect other daily behaviors such as sleep and feeding. Interestingly, the most highly upregulated gene in BMAL1 aKO is *Fabp7*, which influences sleep in humans and mice^58^, as well as long-term memory in *Drosophila*^59^. Thus, further investigation into how astrocytic BMAL1 influences sleep, activity rhythms, or other astrocyte functions such as gliotransmission may uncover non-Aβ related contributions to AD progression.

In conclusion, it is tempting to reduce the complexity of astrocyte activation in AD down to an overall “good” or “bad” effect. Our data, when considered with previous studies, suggests that the answer is more complex, and that astrocyte activation can have different effects on plaque burden depending on the mode of activation and the resulting transcriptional profile. Our findings show that loss of astrocytic *Bmal1* enhances plaque-related astrocyte activation and alters gene expression but does not alter plaque burden in two different mouse models. These findings provide insights into the effects of glial circadian clock disruption in AD and provide future opportunity to define astrocyte activation states at the molecular level that may impart protective, neutral, or destructive effects in the AD brain.

## Supplementary Information

Supplementary Information.
