# Supplementary material for: Astrocytes deficient in circadian clock gene Bmal1 show enhanced activation responses to amyloid-beta pathology without changing plaque burden
Source: Sci Rep. 2022 Feb 2;12:1796. doi: 10.1038/s41598-022-05862-z (PMC8810760; doi:10.1038/s41598-022-05862-z)
Supplement: Supplementary file 1 — Supplementary Information. [file 41598_2022_5862_MOESM1_ESM.pdf]

Supplemental Figure 1.

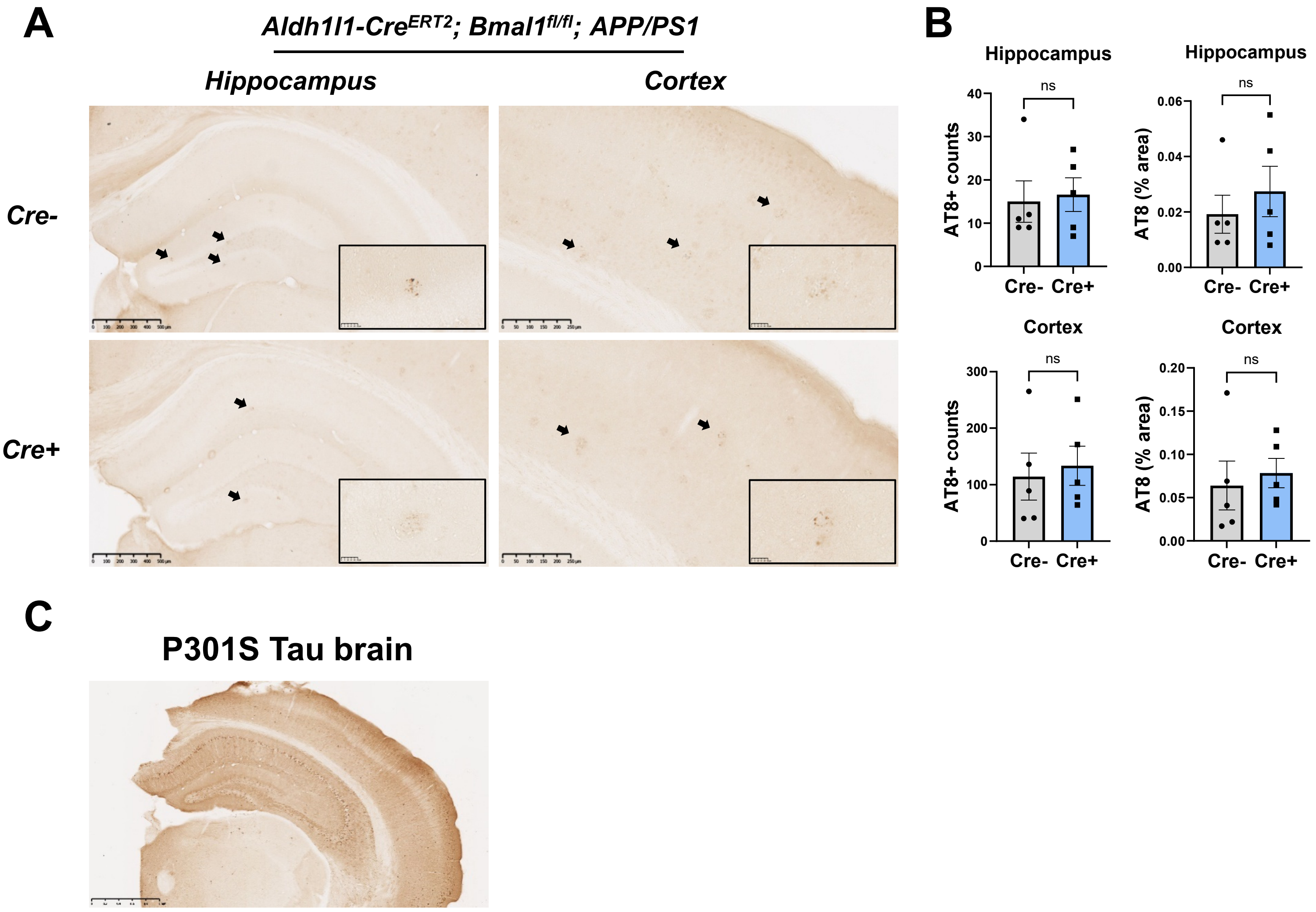

Supplemental Figure 1: *Bmal1* deletion in astrocytes does not affect tau phosphorylation in APP/PS1 mice. A) AT8 staining for phosphorylated tau in brain sections from 4-month-old BMAL1 aKO; APP/PS1-21 mice and Cre- controls. Arrows in low magnification images indicate clusters of AT8 signal around plaques (hippocampus scale bar= 500µm, cortex image scale bar= 250µm). Insets show clusters of AT8 signal (scale bar=25µm). B) Quantifications of AT8 particle counts and percent area per hippocampus or cortex (n=5 mice per group). C) AT8 staining from P301S tau brain as positive control stained alongside the APP/PS1-21 brains, indicating much greater AT8 signal than in APP/PS1-21 brain (scale bar= 1mm).

Supplemental Figure 2.

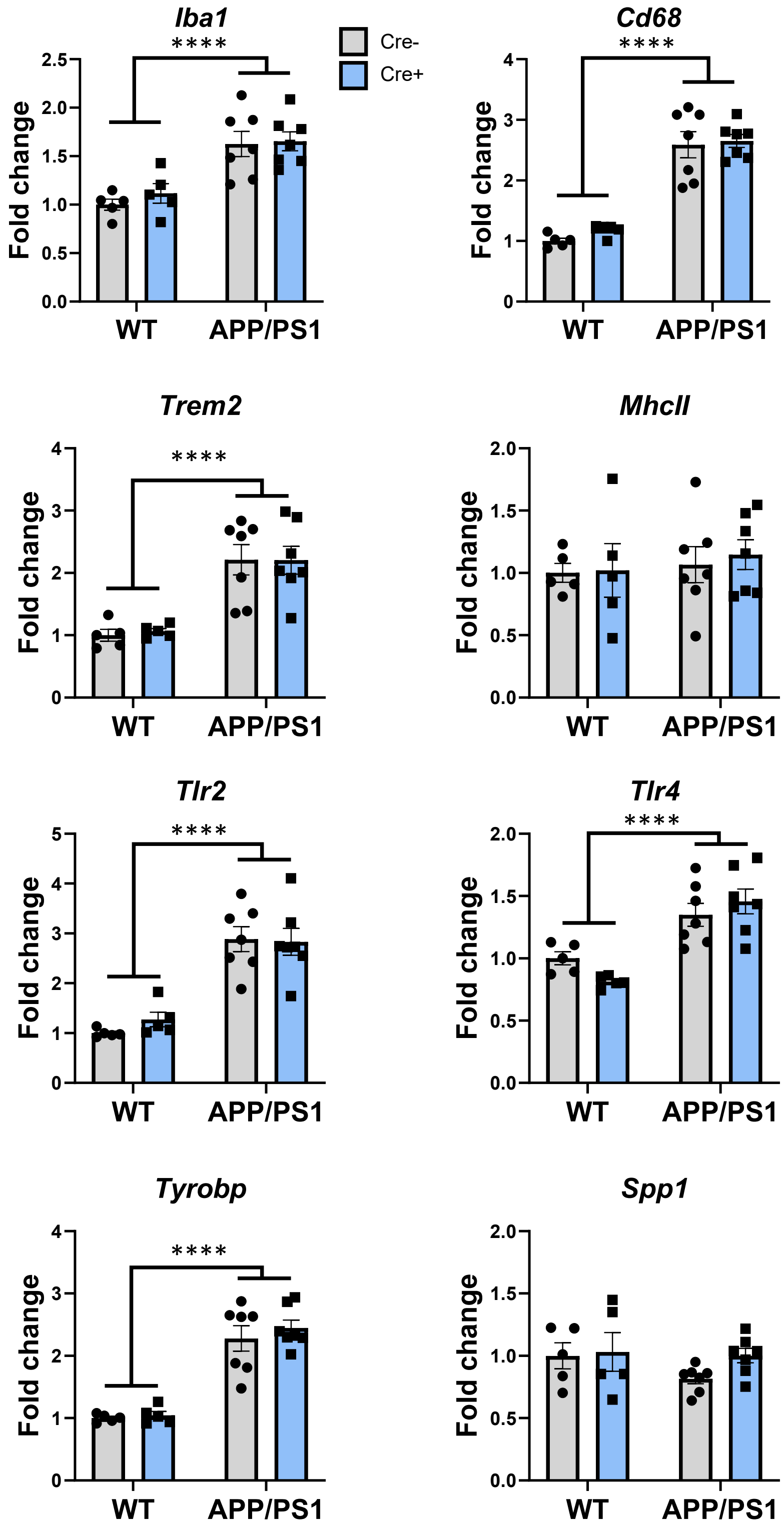

Supplemental Figure 2: APP/PS1 induces microglial activation, which is not influenced by BMAL1 aKO. A) Fluidigm qPCR gene expression analysis of microglial genes in cortex tissue from BMAL1 aKO; APP/PS1-21 and Cre- controls given tamoxifen at 2 months and harvested at 4 months of age. Blue bars indicate Cre+ BMAL1 aKO mice, grey are Cre- controls. N=5-7 mice per group, ****=p<0.0001 by two-way ANOVA with Sidak multiple comparisons test.
